# A rare case report of a saddle pulmonary embolism presenting with high grade fevers, responsive to anticoagulation

**DOI:** 10.1097/MD.0000000000010002

**Published:** 2018-03-02

**Authors:** Muhammad Saad, Danial H. Shaikh, Muhammad Adrish

**Affiliations:** a Department of Internal Medicine; b Division of Pulmonary and Critical Care, Department of Medicine, BronxCare Hospital Center, Affiliated with Icahn School of Medicine at Mount Sinai, New York.

**Keywords:** anticoagulation, fever of unknown origin, fever in pulmonary embolism, high grade fever, pulmonary embolism, saddle embolus, unknown fever

## Abstract

**Rationale:**

Pulmonary embolism can manifest by a myriad of clinical symptoms. High grade fever is a rare presentation of thromboembolic phenomenon.

**Patient concerns:**

A middle aged woman presented with high grade fevers.

**Diagnoses:**

Patient remained febrile despite broad spectrum antibiotics. All cultures were negative. CT angiogram of the chest was done, eliciting a large saddle embolus.

**Interventions:**

Intravenous tissue plasminogen activator (t-PA) was administered and subsequently started on anticoagulation. Patient became afebrile 3 days after initiation of anticoagulation and all antibiotics were discontinued.

**Outcomes:**

We demonstrate a case of a saddle pulmonary embolism presenting with high grade fevers that responded to anticoagulation.

**Lessons:**

It is imperative to include pulmonary embolism in the differential diagnosis, when presented with high-grade fever in patients with unclear diagnosis.

## 1 Introduction

Pulmonary embolism (PE) can manifest by a myriad of clinical symptoms including cough, pleuritic chest pain, and hemoptysis. High grade fever (>39 °C) is a rare presentation of thromboembolic phenomenon observed in only 16% of the patients.^[1]^ Although, high grade fever has no impact on disease severity or prognosis, its recognition can lead to cost-effectiveness and high value care. Clinicians should suspect pulmonary embolism as a cause of fever in view of negative cultures and antibiotic ineffectiveness in appropriate clinical setting.^[2]^ We demonstrate a case of a saddle pulmonary embolism presenting with high grade fever that responded to anticoagulation.

## 2 Case presentation

A woman in her 40s, presented to the emergency department with complains of nausea, vomiting, and dizziness for the last 2 days. She was experiencing non-bilious, non-bloody, and non-projectile vomiting associated with diffuse abdominal pain and non-bloody watery diarrhea. Patient reported subjective fever associated with rigors and chills but denied diurnal variation, any sick contacts, uncooked/stale food consumption, recent travels, or any urinary symptoms. Her comorbid conditions included human immunodeficiency virus (HIV) infection with a recent CD4+ count of 810 cells/μL (acquired through unknown source, compliant on antiretroviral therapy), subclinical hypothyroidism, hypertension, and diabetes mellitus. She had tubal ligation done 5 years ago and did not report any family history of cancers, heart, or lung diseases. She was non-smoker, non-alcoholic, and had never used recreational drugs or herbal medications. She worked as a home attendant and had 3 children, lived in United States for the past 15 years with last travel to Africa 6 years ago.

**Figure 1:**
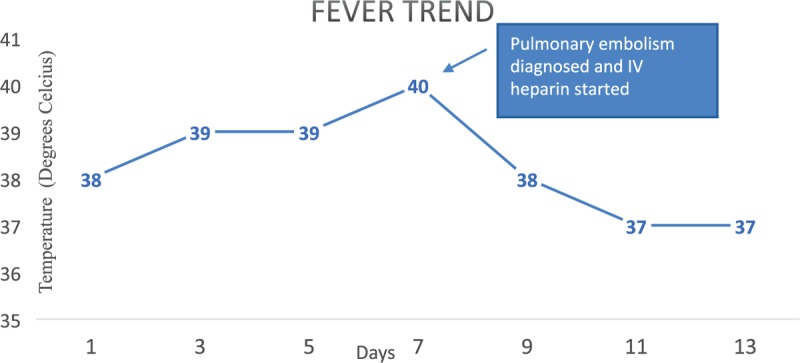
Graphical representation of patient's fever curve in Celsius (*y*-axis) against time in days (*x*-axis).

On presentation to the emergency department, patient was found to be febrile with a temperature of 38 °C (Fig. 1), tachycardia of 112 beats/min, blood pressure of 125/75 mmHg, and an oxygen saturation of 100% on room air. She was lethargic but alert and oriented and not in an acute distress. Cardiovascular exam was unremarkable except for tachycardia. Rest of the physical examination was unremarkable. Initial laboratory findings are shown in Tables 1 and 2. Patient was started on intravenous fluids and intravenous antibiotics for suspected acute cholangitis. Computed tomography (CT) scan of the abdomen was performed to discern cause of sepsis, results of which were inconclusive. Advance imaging including magnetic resonance cholangiopancreatography (MRCP) was negative for any intrahepatic, biliary, or pancreatic ductal pathology. She continued to have persistent fevers despite sufficient antibiotics, and all cultures remained negative. Her liver function tests normalized within 3 days, without any intervention. Extensive work up for fever including testing for legionella, mycoplasma, leptospirosis, herpes simplex, Cytomegalovirus (CMV), strongyloides, stool ova and parasites were negative.

**Table 1:**
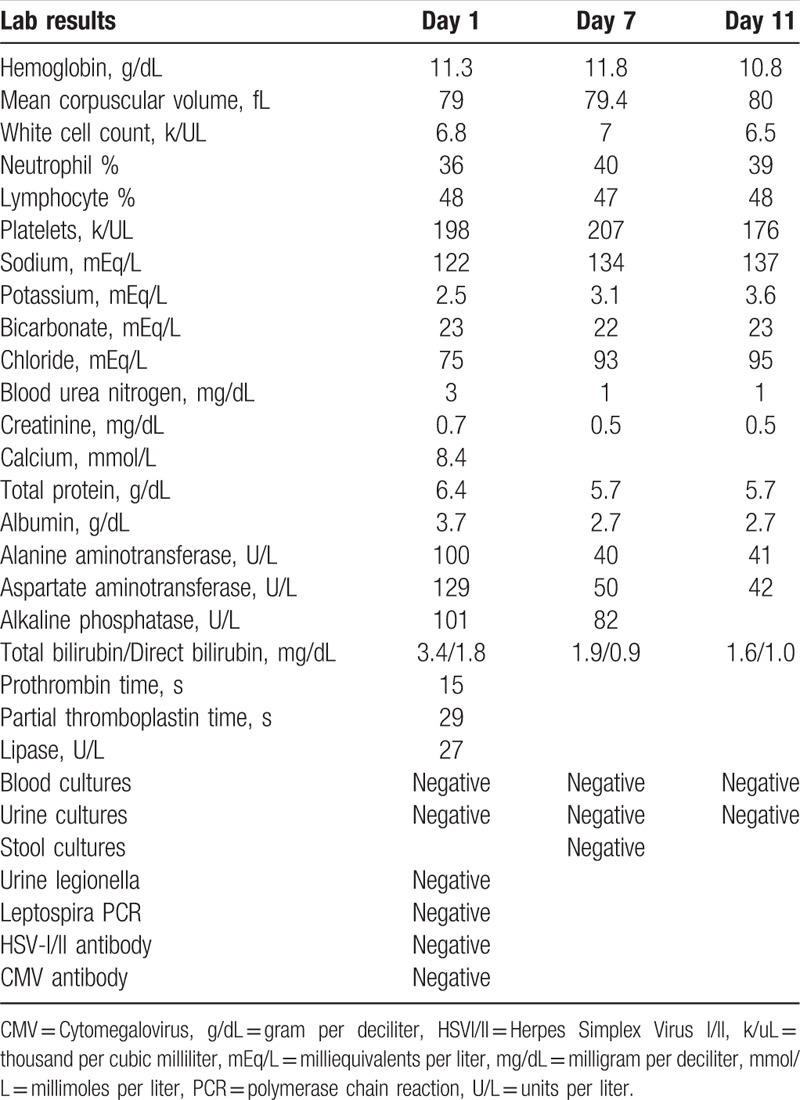
Laboratory data with trend.

**Table 2:**
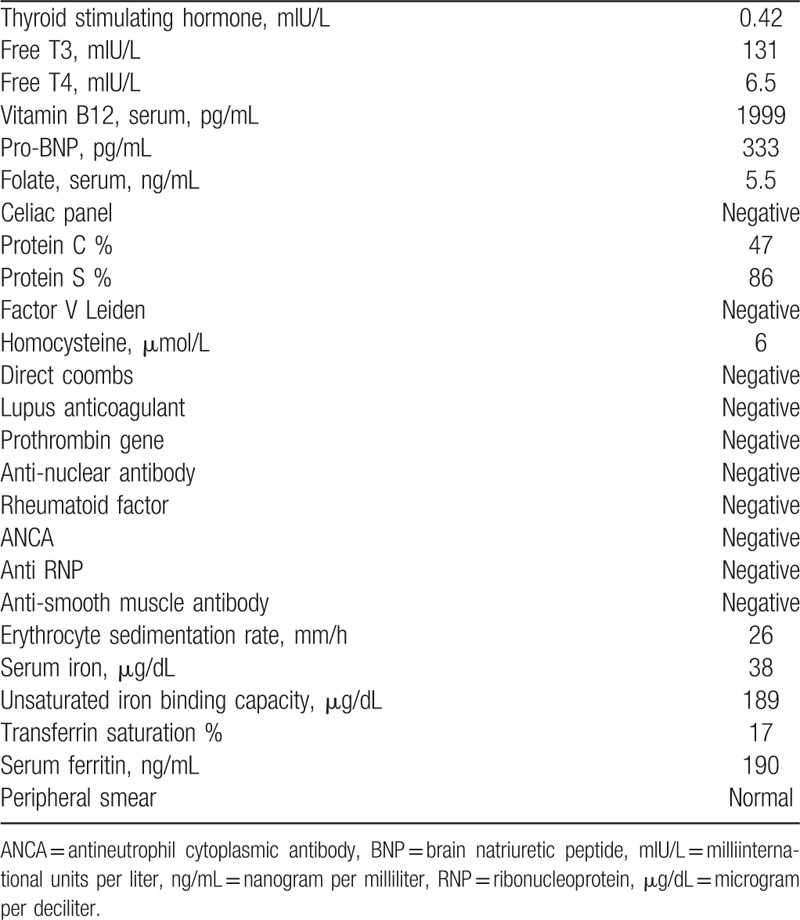
Miscellaneous laboratory data.

**Figure 2:**
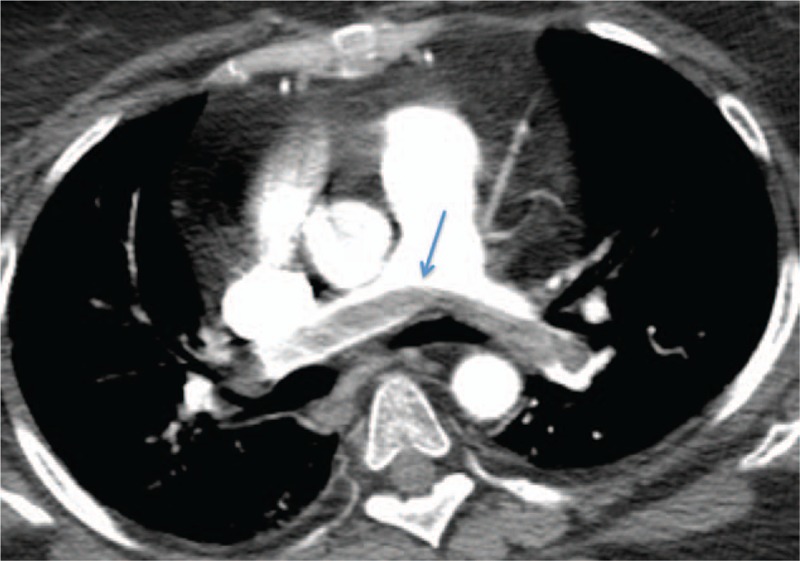
CT angiogram of the chest showing saddle embolus in pulmonary arteries (marked with blue arrow). CT = computed tomography.

**Figure 3:**
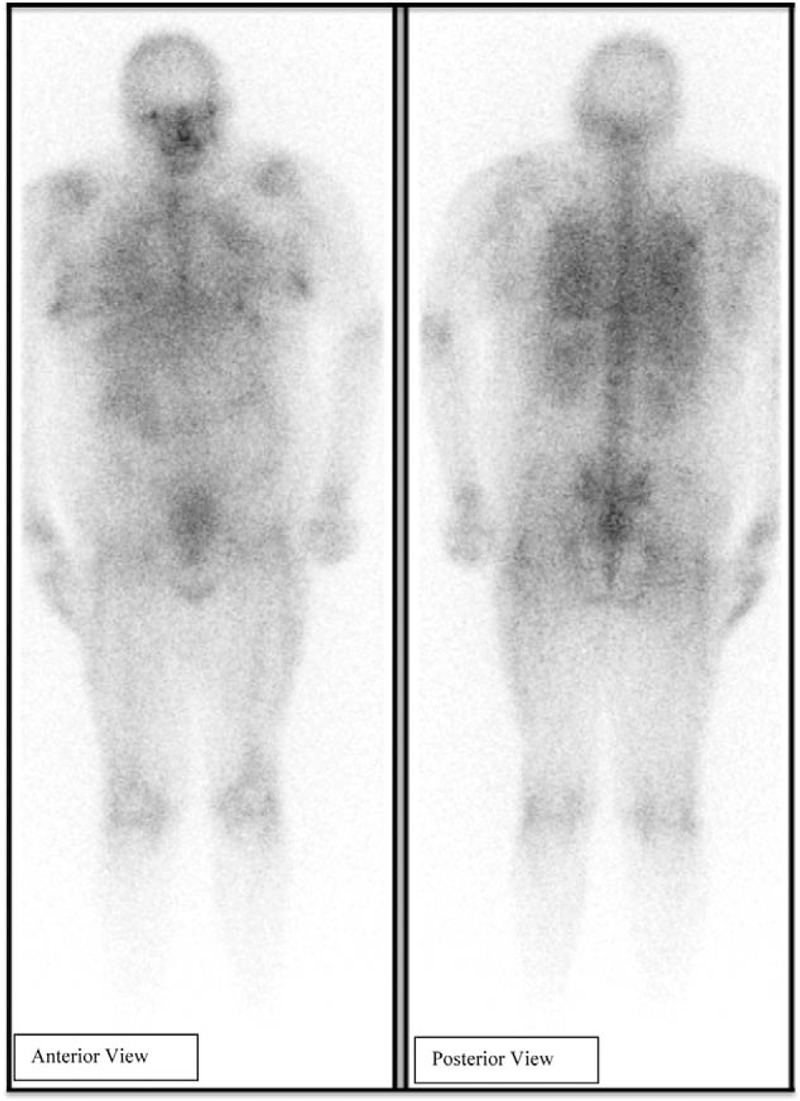
Gallium scan showing diffuse uptake in bilateral lungs.

On day 7 of Intensive Care Unit (ICU) stay, patient became tachypneic and hypotensive, with oxygen saturation dropping to 88%. She was started on supplemental oxygen via nasal cannula and a bedside goal-directed echocardiography was performed by the ICU team. Echocardiography was significant for a right ventricular strain pattern. An emergent CT angiogram of the chest was done, eliciting a large saddle embolus (Fig. 2). Ultrasound of the lower extremities showed right sided superficial femoral and popliteal vein deep vein thrombosis (DVT). Intravenous tissue plasminogen activator (t-PA) was administered and later the patient was started on intravenous heparin infusion. On day 9, Gallium scan was done (Fig. 3) that showed uptake exclusively in the lungs, and confirmed suspicion of pulmonary embolism as the sole cause of her fever. All antibiotics were discontinued and patient eventually became afebrile on day 11 of ICU stay (3 days after initiation of anticoagulation). She was observed for continued stability and transferred to the floor for further care.

## 3 Discussion

Fever has long been recognized as an accompanying sign of pulmonary embolism. Roughly half of all patients with PE and DVT are noted to have fevers ranging from 38 to 38.5 °C.^[1,2]^ Demonstrated through studies, as early as the 1950s, Israel and Goldstein^[3]^ observed low-grade fevers in 78.9% of patients, despite the use of antibiotics. Murray et al,^[4]^ in the late 70s, showed evidence of fever exceeding 38 °C in 57.1% of patients suffering from acute PE that could not be explained by any other cause. Stein et al^[5]^ documented temperatures >37.5 °C in 50% of their patients with acute pulmonary embolism, but could not clearly define causality of the fever. Most recently, observed in prospective investigation of pulmonary embolism diagnosis (PIOPED), a large prospective, multicenter, comparative study, fever without any other definite or possible explanatory cause was observed in 14% of pulmonary embolism patients, of which 6% had high grade fever (>39 °C).^[2,5,6]^

The etiology of fever in PE has largely remained unknown. Several experts have speculated and attributed the pathogenesis to factors such as local inflammation secondary to vascular irritation and/or the release of chemotactic factors. The latter being a phenomenon of tissue injury/complement activation.^[7]^ Others still, have hypothesized that mechanism behind fever in PE is pulmonary infarction with resultant tissue necrosis, hemorrhage with extravagated blood, atelectasis or self-limited, occult super infection.^[4,8]^

Previously, it was reported in literature that if the temperature is >39 °C, the diagnosis of pulmonary embolism is unlikely.^[9]^ However, newer data have since revised such statements. This is in part due to a handful of studies, over decades, demonstrating fevers >39 °C in patients with acute pulmonary embolism.^[4,5,8–11]^

A challenge presented to clinicians managing PE with fevers is to exclude an infectious etiology such as community acquired pneumonia, since both may present as fevers with accompanying chest x-ray abnormalities and similar leukocyte counts. Physician apprehension towards fevers leads to unwarranted use of antibiotics, at times targeting the fever alone. Excessive use of antibiotics not only results in increased adverse events, but also increases resistance of microbiota present within the in-patient setting. Antibiotic use in PE is in fact detrimental; with some studies showing higher 7th-day body temperature in patients with PE receiving antibiotics compared with those that did not.^[1,5,12,13]^ These findings suggested that adding antibiotics provided no benefit in PE patients. Infarction was also more frequently noted in the group of PE patients who received antibiotics versus those who did not.^[8]^ Usually fever in pulmonary embolism responds well with initiation of anticoagulation with resolution achieved within 72 hours in majority of the cases.^[14,15]^ Rarely, drug related fever has been a confounding differential in final diagnosis. Early mobilization is the key towards better outcome along with anticoagulation.^[2,4,16,17]^

## 4 Conclusion

Persistent high-grade fevers in pulmonary embolism remain a seldom reported, rare entity. It is imperative to include PE in the differentials, when presented with high-grade fevers, in a patient where the diagnosis is unclear. We suggest that fever cessation after initiation of anticoagulation therapy points towards fever secondary to PE, and deserves further investigation.
